# Draft Genome Sequence for the Symbiotic Pine Mushroom Tricholoma matsutake

**DOI:** 10.1128/mra.01271-22

**Published:** 2023-05-03

**Authors:** Min-Jeong Kang, Eun-Kyung Bae, Eung-Jun Park, Kang-Hyeon Ka, Mi-Ra Son, Ki-Tae Kim, Jei-Wan Lee

**Affiliations:** a Forest Bioresources Department, National Institute of Forest Science, Suwon, Republic of Korea; b Department of Agricultural Life Science, Sunchon National University, Suncheon, Republic of Korea; University of California, Riverside

## Abstract

We report the high-quality genome sequence of Tricholoma matsutake strain 2001, which was isolated from a mushroom fruiting body in South Korea. The genome has 80 contigs, a size of 162.6 Mb, and an *N*_50_ value of 5,103,859 bp and will provide insight into the symbiotic association between T. matsutake and Pinus densiflora.

## ANNOUNCEMENT

The production of Tricholoma matsutake, a symbiotic pine mushroom, has declined due to climate change and newly occurring pine tree diseases (1, 2), emphasizing the need to understand the mycorrhizal symbiosis with coniferous trees (3, 4). Although genomes of T. matsutake have been sequenced (5–7) (Table 1), they are highly fragmented, limiting in-depth molecular analysis.

**TABLE 1:** Genome assembly statistics for Tricholoma matsutake strain 2001 and the previously reported strains from GenBank

Parameter	Data for strain				
	NIFoS 2001	945	KMCC04578	MG52	NBRC 30605
Genome assembly size (Mbp)	162.6	175.8	189.0	155.0	131.7
No. of scaffolds	80	2,545	5,255	58,276	88,884
*N*_50_ (bp)	5,103,859	320,875	93,406	9,294	2,909
*L*_50_	10	167	589	3,971	9,526
GC content (%)	45.50	39.27	34.41	44.52	45.28
No. of genes	15,947	23,170	15,305	22,912	NA
Sequencing technology	PacBio, Illumina	Illumina	Illumina	Illumina	Illumina
WGS project no.	JALPZM01	WIUY01	PKSN02	QMFF01	BDDP01
Whole-genome accession no.	JALPZM000000000	WIUY00000000	PKSN00000000	QMFF00000000	BDDP00000000
Isolation location	South Korea	NA^*a*^	South Korea	China	Japan
Reference or source	This study	7	6	5	Unpublished

a NA, not available.

T. matsutake strain 2001 was isolated from a fruiting body in Yeongwol-gun, South Korea, in September 2012. The strain was stored at the National Institute of Forest Science (NIFoS) stock center and was one of the strains showing the best mycorrhization rate (8). The fungal isolate was cultured for 38 days at 25°C on potato dextrose agar (PDA) and identified by sequencing of the internal transcribed spacer (ITS), which was amplified using the universal primers ITS1 and ITS4 (9).

Genomic DNA was extracted using the DNeasy plant minikit (Qiagen, Hilden, Germany) following the manufacturer’s protocol and evaluated by 1% agarose gel electrophoresis and the Qubit double-stranded DNA (dsDNA) high-sensitivity (HS) assay kit (Thermo Fisher Scientific, Eugene, OR, USA). The genomic DNA was sheared using a g-TUBE (Covaris, Woburn, MA, USA), and small fragments were removed using the BluePippin size selection system (Sage Science, Beverly, MA, USA). The size-selected library, with an average size of 20 kbp, was prepared with the SMRTbell DNA library construction protocol and sequenced using the Pacific Biosciences (PacBio) Sequel platform (DNA Link, Seoul, South Korea). This long-read sequencing produced 2,017,716 subreads (21,386,538,649 nucleotides [nt]), with a read *N*_50_ value of 19,155 nt. The subreads were filtered with single-molecule real-time (SMRT) Link v8.0 with default parameters, and the SMRTbell adapters were trimmed on the PacBio Sequel instrument.

Short-read sequencing on the Illumina NovaSeq 6000 platform was used to correct errors in the long-read assembly (DNA Link). The DNA sample quality was evaluated using 1% agarose gel electrophoresis and the Qubit dsDNA HS assay kit (Thermo Fisher Scientific). The DNA library was prepared using the Illumina TruSeq Nano DNA library preparation protocol, in which high-molecular-weight genomic DNA was randomly sheared and processed by blunt-ending, phosphorylation, and adapter ligation at both ends. Ligated DNA was PCR amplified, and the quality of the libraries was verified using a 2100 Bioanalyzer (Agilent Technologies, Santa Clara, CA, USA). After quantitative PCR (qPCR) using SYBR Green PCR Master Mix (Applied Biosystems, Waltham, MA, USA), indexed libraries were combined in equimolar amounts and subjected to whole-genome sequencing (WGS) with the 2 × 150-bp sequencing protocol. The sample was sequenced, with a total size of 14,191,932,810 bp and 93,986,310 reads.

The genome was assembled using NextDenovo v2.4.0 with the long-read sequences. NextPolish v1.3.1 was used for error correction with default parameters (10). The genome assembly was checked using Benchmarking Universal Single-Copy Orthologs (BUSCO) v5.3.1 with the fungi_odb10.2019-11-20 data set (11).

The genome of T. matsutake strain 2001 has 80 contigs, a size of 162.6 Mb, a GC content of 45.5%, and an *N*_50_ value of 5,103,859 bp (Table 1). Of the total 758-BUSCO data set searched, our assembly displayed 712 complete BUSCOs (approximately 93.9%), with 703 being single copy (92.7%). This high-quality genome of T. matsutake will provide insight into the symbiotic association between T. matsutake and Pinus densiflora and allow future studies using genomic approaches.

### Data availability.

The genome data generated in this study were submitted to NCBI with GenBank assembly accession number GCA_026213095.1, WGS accession number JALPZM000000000, BioProject accession number PRJNA831907, and BioSample accession number SAMN27770844.

## References

[1] An G-H, Cho J-H, Kim O-T, Han J-H. 2021. Metagenomic analysis of bacterial and fungal communities inhabiting Shiro dominant soils of two production regions of *Tricholoma Matsutake* S. Ito & S. Imai in Korea. Forests 12:758. doi:10.3390/f12060758.

[2] Yamanaka T, Yamada A, Furukawa H. 2020. Advances in the cultivation of the highly-prized ectomycorrhizal mushroom *Tricholoma matsutake*. Mycoscience 61:49–57. doi:10.1016/j.myc.2020.01.001.

[3] Oh S-Y, Lim YW. 2018. Root-associated bacteria influencing mycelial growth of *Tricholoma matsutake* (pine mushroom). J Microbiol 56:399–407. doi:10.1007/s12275-018-7491-y. 29858828

[4] Park KH, Oh SY, Yoo S, Park MS, Fong JJ, Lim YW. 2020. Successional change of the fungal microbiome pine seedling roots inoculated with *Tricholoma matsutake*. Front Microbiol 11:574146. doi:10.3389/fmicb.2020.574146. 33101248 PMC7545793

[5] Li H, Wu S, Ma X, Chen W, Zhang J, Duan S, Gao Y, Kui L, Huang W, Wu P, Shi R, Li Y, Wang Y, Li J, Guo X, Luo X, Li Q, Xiong C, Liu H, Gui M, Sheng J, Dong Y. 2018. The genome sequences of 90 mushrooms. Sci Rep 8:9982. doi:10.1038/s41598-018-28303-2. 29967427 PMC6028375

[6] Min B, Yoon H, Park J, Oh YL, Kong WS, Kim JG, Choi IG. 2020. Unusual genome expansion and transcription suppression in ectomycorrhizal *Tricholoma matsutake* by insertions of transposable elements. PLoS One 15:e0227923. doi:10.1371/journal.pone.0227923. 31978083 PMC6980582

[7] Miyauchi S, Kiss E, Kuo A, Drula E, Kohler A, Sánchez-García M, Morin E, Andreopoulos B, Barry KW, Bonito G, Buée M, Carver A, Chen C, Cichocki N, Clum A, Culley D, Crous PW, Fauchery L, Girlanda M, Hayes RD, Kéri Z, LaButti K, Lipzen A, Lombard V, Magnuson J, Maillard F, Murat C, Nolan M, Ohm RA, Pangilinan J, Pereira MF, Perotto S, Peter M, Pfister S, Riley R, Sitrit Y, Stielow JB, Szöllősi G, Žifčáková L, Štursová M, Spatafora JW, Tedersoo L, Vaario LM, Yamada A, Yan M, Wang P, Xu J, Bruns T, Baldrian P, Vilgalys R, Dunand C, Henrissat B, Grigoriev IV, Hibbett D, Nagy LG, Martin FM. 2020. Large-scale genome sequencing of mycorrhizal fungi provides insights into the early evolution of symbiotic traits. Nat Commun 11:5125. doi:10.1038/s41467-020-18795-w. 33046698 PMC7550596

[8] Jeon S-M, Ka K-H. 2016. Korean *Tricholoma matsutake* strains that promote mycorrhization and growth of *Pinus densiflora* seedlings. Kor J Mycol 44:155–165.

[9] White TJ, Bruns T, Lee S, Taylor J. 1990. Amplification and direct sequencing of fungal ribosomal RNA genes for phylogenetics. In PCR protocols: a guide to methods and applications. Academic Press, New York, NY.

[10] Hu J, Fan J, Sun Z, Liu S. 2020. NextPolish: a fast and efficient genome polishing tool for long-read assembly. Bioinformatics 36:2253–2255. doi:10.1093/bioinformatics/btz891. 31778144

[11] Manni M, Berkeley MR, Seppey M, Zdobnov EM. 2021. BUSCO: assessing genomic data quality and beyond. Curr Protoc 1:e323. doi:10.1002/cpz1.323. 34936221

